# Prewetting Induced Hydrophilicity to Augment Photocatalytic Activity of Nanocalcite @ Polyester Fabric

**DOI:** 10.3390/polym14020295

**Published:** 2022-01-12

**Authors:** Ayesha Qayyum, Ijaz Ahmad Bhatti, Ambreen Ashar, Asim Jilani, Javed Iqbal, Muhammad Mohsin, Tehmeena Ishaq, Shabbir Muhammad, S. Wageh, Mohsin Raza Dustgeer

**Affiliations:** 1 Department of Chemistry, University of Agriculture Faisalabad (UAF), Punjab 38040, Pakistan; ashichaudry786@gmail.com (A.Q.); ijazchem@yahoo.com (I.A.B.); ishaqtehmeena@gmail.com (T.I.); 2 Center of Nanotechnology, King Abdul-Aziz University, Jeddah 21589, Saudi Arabia; iqbaljavedch@gmail.com; 3 Department of Chemistry, Sargodha Campus, The University of Lahore, Sargodha 40100, Pakistan; 4 Research Center for Advanced Materials Science (RCAMS), King Khalid University, Abha 61413, Saudi Arabia; mshabbir@kku.edu.sa; 5 Department of Physics, College of Science, King Khalid University, Abha 61413, Saudi Arabia; 6 Department of Physics, Faculty of Science, King Abdulaziz University, Jeddah 21589, Saudi Arabia; wageh1@yahoo.com; 7 Physics and Engineering Mathematics Department, Faculty of Electronic Engineering, Menoufia University, Menouf 32952, Egypt; 8 Department of Environmental Sciences and Engineering, Government College University Faisalabad, Faisalabad 38000, Pakistan; dustgeerraza@gmail.com

**Keywords:** nanocalcite, polyester fabric, acidic and basic prewetting, hydrophilicity, photocatalytic activity

## Abstract

To eliminate imidacloprid insecticide from wastewater, nanocalcite was grafted onto the surface of pretreated polyester fabric. The process of seeding was followed by the low temperature hydrothermal method for the growth of nanocalcite for the functionalization of fabric. The goal of this study was to improve the hydrophilicity of the nanocalcite photocatalyst that had been grafted onto the surface of polyester fabric (PF) using acidic and basic prewetting techniques. The morphological characteristics, crystalline nature, surface charge density, functional groups of surface-modified nanocalcite @ PF were determined via SEM, XRD, FTIR, and Zeta potential (ZP), respectively. Characterization results critically disclosed surface roughness due to excessive induction of hydroxyl groups, rhombohedral crystal structure, and high charge density (0.721 mS/cm). Moreover, contact angle of nanocalcite @ PF was calculated to be 137.54° while after acidic and basic prewetting, it was reduced to 87.17° and 48.19°. Similarly, bandgap of the as fabricated nanocalcite was found to be 3.5 eV, while basic prewetted PF showed a reduction in band gap (2.9 eV). The solar photocatalytic mineralization of imidacloprid as a probe pollutant was used to assess the improvement in photocatalytic activity of nanocalcite @ PF after prewetting. Response surface methodology was used to statistically optimize the solar exposure time, concentration of the oxidant, and initial pH of the reaction mixture. Maximum solar photocatalytic degradation of the imidacloprid was achieved by basic prewetted nanocalcite @ PF (up to 91.49%), which was superior to acidic prewetted fabric and as-fabricated nanocalcite @ PF. Furthermore, HPLC and FTIR findings further indicated that imidacloprid was decomposed vastly to harmless species by basic prewetted nanocalcite @ PF.

## 1. Introduction

Polyethylene terephthalate (PET) is one of the most widely used synthetic textiles. Such fabrics have many advantages such as washability, abrasion resistance high strength, dimensional stability, wrinkle resistance, attractive handling, and stretch resistance. On the other hand, some unwanted features have emerged such as the accumulation of static electric charge and a lack of hydrophilicity due to a poor moisture recovery capacity (0.4 percent) [1,2,3,4,5]. Polyester has been widely used as an apparel and technical textile material in the form of fabrics, films, and plastics due to its excellent mechanical and physical properties [6,7]. Moreover, polyester fabric has flexible but robust mechanical properties, rendering polyester fabric a suitable substrate to be used as an optimal candidate for oil/water separation [8,9]. Since the polyester fabric is highly inert with low surface energy, it has an appropriate functional group providing suitable chemical features to the surface. Several surface modification methods for polyester have been reported such as photo-induced irradiation, electron beam irradiation, enzymatic modification, and plasma treatments [10]. Wetting property of fibrous material by a liquid is vital to their functional performance and chemical processing [11,12]. For decades, fibrous material has been used for surface coating due to its special characteristics such as its occurrence in abundance, renewable origin, relatively high specific strength and modulus, light-weight, low cost, and biodegradability [13,14]. With the recent advances in nanotechnology and materials engineering, different functionalities can now be incorporated into textile fabric [15,16]. Many nanometal oxides have been reported to be immobilized onto the surface of the polyester fabric. Grafting of the surface of polyester fabric with nanometal oxides enhance the surface area due to their small size and uniform coverage [17,18]. Metal oxides have been employed as surface coatings due to their applications in self-cleaning coatings, anti-abrasion, defogging, antistatic, and anti-reflection.

Heterogeneous photocatalysis is a surface phenomenon that can play an important role in the purification of water [19]. Many water pollutants have been reported to act as toxic chemicals. Persistent organic pollutants (POPs) are a group of chemical compounds of different origins and have common characteristics such as semi volatility, hydrophobicity, high toxicity, and continue to exist in the environment. The presence of POPs in textile effluents leads to a series of negative effects on the ecology [20,21,22]. Insecticides are used in households, agriculture, medical industry, etc., which enter into water sources, causing toxicity in the aquatic and terrestrial organisms, leading to alteration of the ecosystem, in addition to human health [23,24]. Photocatalytic degradation of organic pollutants by metal oxides has gained attention among researchers due to its efficacy in attacking contaminants of the environment [20,25,26]. Therefore, the efficiency of visible light-mediated photocatalysis is increasing in context to its significance for practical applications in the future. The mechanism of the photocatalyst generates a photogenerated hole-electron pair, which is enhanced by the hydrophilicity, a unique feature of the surface. Hashimoto and co-workers investigated the hydrophilicity mechanisms and suggested that the photogenerated hole-electron pair are trapped on the surface of the metal oxide at the lattice oxygen [27]. The trapped holes are liable to form a weak bond between the lattice oxygen and a metal atom results in producing oxygen vacancies (OV) in the crystal by liberation of oxygen. The oxygen vacancies and hydroxyl group functionalities on its surface promote both hydrophilicity and photocatalytic activity [28]. Photo-reactivity enhanced by OV-induced localized states, which can extend the response to visible irradiation and efficiently trap charge carriers [29]. Hydrophilic and hydrophobic characteristics depend upon the contact angle; for a hydrophilic surface, the water droplet contact angle is <90° and for a hydrophobic surface, it is >90°. Hydrophilicity of the surface can be altered by prewetting pH, which has been reported for surfaces treated with acids (hydrochloric acid and nitric acid) and bases (ammonium hydroxide, potassium hydroxide, and sodium hydroxide) [30,31].

Enhancement of photocatalytic activity through hydrophilicity by a prewetting strategy is a good way to modify nanocalcite @ PF. In this attempt, the modification of the surface hydrophilicity was investigated via prewetting at different pH values, which strongly affected the surface transformation. The effect of surface roughness on the hydrophilic and solar photocatalytic activity of the surface of nanocalcite @ PF was investigated using Imidacloprid insecticide as the probe pollutant.

We used acid and basic prewetting techniques for the enhancement of hydrophilicity of nanocalcite functionalized polyester fabric. The prewetting pH technique itself is unique research as literature available in this context is rare [30]. The untreated surface of nanocalcite @ PF has been characterized by a water contact angle of 137.54°, indicating a hydrophobic surface. The contact angle decreased to 87.19° after acidic, while basic surface prewetting of nanocalcite @ PF yielded a water contact angle of 48.195°. Thus, modification of the surface grafted nanocalcite @ PF after acid–base prewetting significantly affected the surface hydrophilicity. The enhanced hydrophilicity augmented acceleration of the solar photocatalytic reaction for the degradation of pesticide. It is economically feasible to fabricate a prewetted solar photocatalytic reactor with a favorable surface modification to eliminate toxic water pollutants. Reusability of treated water by solar photocatalysis is an advance avenue to cope with the scarcity of water.

## 2. Methodology

### 2.1. Chemicals

Pristine polyester fabric was obtained from the National Textile University, Faisalabad, Pakistan and utilized for grafting nanocalcite and investigation regarding the induction of hydrophilicity. All the reagents used were of analytical grade (Sigma Aldrich, St. Louis, MO, USA) including calcium chloride dihydrate (99.9%), calcium acetate dehydrate (99.9%), potassium hydroxide (99.9%), hydrochloric acid (37%), hydrogen peroxide (35%), and methanol (99%)

### 2.2. Functionalization of Polyester with Nanocalcite

Nanocalcite was grafted onto the surface of polyester, after pretreatment. The process of seeding the pretreated polyester with seeds of calcite was followed by the growth of calcite crystals onto the surface of seeded polyester.

#### 2.2.1. Pretreatment of Polyester

The untreated hydrophobic polyester fabric was pretreated with 2 M aqueous solution of sodium hydroxide for 30 min at 60 °C prior to proceeding to functionalization with nanocalcite. The pretreated fabric after the generation of surface polar groups was rinsed with acetone and ethanol solution at room temperature for 15 min, and then repeatedly washed with distilled water.

#### 2.2.2. Seeding of Polyester with Nanocalcite

The pre-treated fabric was uniformly covered with seeds of nanocalcite by dipping the fabric in seed solution followed by padding and curing at 120 °C for 5 min for each cycle. The seed solution was prepared by refluxing 75 mmol methanolic calcium acetate dihydrate and 75 mmol potassium hydroxide at 60 °C until the seeds appeared. This solution was cooled until the solution became the least cloudy. The seeding cycles were repeated ten times to obtain polyester completely covered with seeds. The procedure was followed to apply the seeds on polyester. The seeded polyester was then cut into small pieces of 30 × 20 cm and further used for the growth of nanocalcite [32].

#### 2.2.3. Growth of Nanocalcite on Polyester

Growth of nanocalcite onto the seeded polyester was executed onto the small pieces of 30 × 20 cm by the low temperature hydrothermal (LTHT) method. To fabricate nanocalcite @ PF, 200 mL of 0.3 M calcium chloride dihydrate (solution A) and 200 mL of 0.4 M potassium hydroxide (solution B) were prepared in DI water. Calcium chloride dihydrate 0.3 M was stirred in a glass beaker of 500 mL capacity at 200 rpm on a magnetic stirrer at room temperature and mixed with 0.4 M potassium hydroxide solution while stirring. This mixture was transferred to a stainless steel jar with a 500 mL capacity along with the piece of seeded polyester. Five such jars were filled with 400 mL of the reaction mixture and a piece of polyester, fitted with heavy lid, screwed tightly and fixed in the socket of the revolving setup dipped in a glycerin bath of a Launder-o-meter (TC-M-25), while the temperature was maintained at 90 °C. After two hours, the contents were cooled down to room temperature and washed with DI water multiple times before finally with ethanol to remove the impurities. These were dried in a hot air oven at 50 °C for two hours and then preserved in plastic bags. No surfactant or capping agent was used in this method [17].

#### 2.2.4. Prewetting of Samples

**Figure 1:**
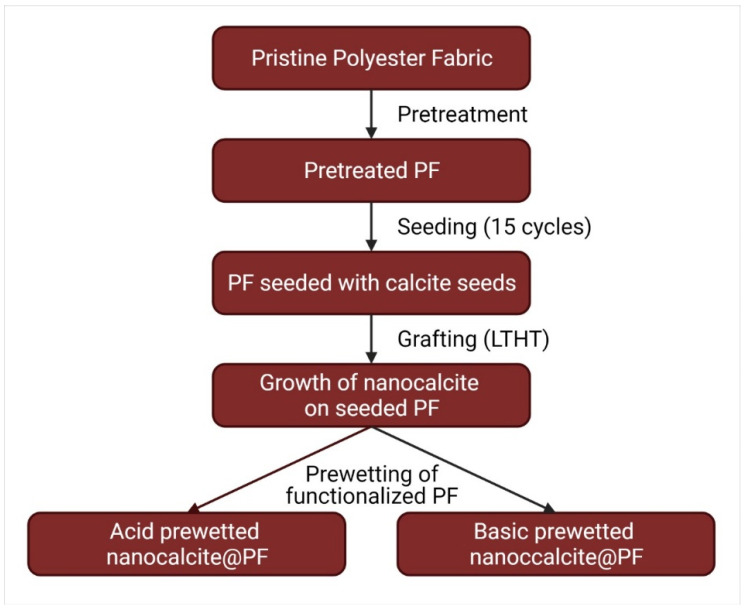
Flowchart of the fabrication of prewetted nanocalcite @ PF.

The pieces of nanocalcite @ PF were cut into swatches of (5 × 5 cm^2^) and the effect of pH prewetting on the hydrophilicity of nanocalcite @ PF were investigated both by acidic and basic prewetting treatments. For the acid and basic prewetting, hydrochloric acid (37% Merck) and potassium hydroxide (99.95% Merck) were used to prepare aqueous solutions of pH 2–13. Flowchart of the fabrication of prewetted nanocalcite @ PF is shown in Figure 1 [30].

### 2.3. Characterization of Samples of Nanocalcite @ PF

The crystallinity of the as-fabricated and prewetted nanocalcite @ PF was determined by XRD (Jeol JDX-3532 diffractometer, Tokyo, Japan) utilizing CuKα irradiation (λ = 1.54 Å). The morphology variations on prewetting were observed by SEM (Quanta 2500. FEG, USA). The specific functional groups that appeared on prewetting were investigated by FTIR (Bruker IFS 125HR, Yokohama, Japan). Modification of the optical properties of nanocalcite @ PF on prewetting was examined by diffused reflectance spectra (Perkin Elmer Lambda 1050, Buckinghamshire, UK) and bandgap calculations. Zeta potential was measured on a Malvern Panalytical (M3-PALS, Malvern, Worcestershire, UK).

### 2.4. Assessment of Comparative Hydrophilicity of Nanocalcite @ PF

To determine the hydrophilicity, the wettability of the surface grafted nanocalcite @ PF was measured by contact angle using optical tensiometers. To define the capability of the fabric to wick moistness, the wicking method was used. The standard wicking test (DIN 53924) involves the cutting of the test sample swatch (5 × 5 cm^2^) and hanging the sample with a rod [33]. In the wicking method, the strip of fabric was hung vertically in distilled water and the wicking height was measured at different time intervals (i.e., 0, 10, 20, 30, 40, and 50 s). As-fabricated and prewetted surface grafted on nanocalcite @ PF were tested by a vertical wicking tester based on DIN 53924 standards [34,35]. The water rose vertically in the fibers of the fabric against gravity due to the effect of capillary action. To find the wickability of the as-fabricated and prewetted fabric pieces, two factors of wicking time and wicking height were measured. The water rising height (in cm) was taken at different time intervals. Contact angle of the as-fabricated, acid and basic prewetted samples of nanocalcite @ PF were measured and their average values reported. The contact angles of the as-fabricated, acidic, and basic prewetted nanocalcite @ PF swatch samples were measured by optical tensiometers A drop of water injected onto the surface grafted nanocalcite @ PF via microinjector syringe (5 µL) pointed vertically down onto the surface of the PF, with a high resolution (60 fps), monochromatic (light source), range of contact angle from 0° to 180°, video camera built into the system to capture the image of the water drop, and then analyzed using analysis software [36,37]. The image of a water droplet was captured by a video camera and the average contact angle was measured using the sessile drop method [38].

### 2.5. Determination of Photocatalytic Activity of Nanocalcite @ PF

Experimentation regarding the photocatalytic activity measurement was carried out in a borosilicate glass container of 10 × 10 × 4 cm^3^ using a 100 mL working solution of imidacloprid for each sample at ambient temperature (30–35 °C). The parameters influencing the photocatalytic degradation reaction of imidacloprid such as the concentration of hydrogen peroxide as the supporting oxidant, pH, and irradiation time were optimized by using response surface methodology (RSM) (Table 1). Percentage degradation of imidacloprid after the photocatalytic treatment was taken as a response and measured by absorption spectra of solutions using the UV/Visible spectroscopic technique. The mathematical relationship provided by the central composite design explained the interaction between three variables (X_1_, X_2_, and X_3_) as the response.

The photocatalytic degradation of imidacloprid was estimated in the month of April under sunlight (flux 1370 watts/m^2^) [39] and average solar flux of ∼600 watts/m^2^ using 5 × 5 cm swatches of nanocalcite @ PF as a photocatalyst under optimized conditions. The swatches of the as-fabricated and prewetted samples of nanocalcite @ PF were suspended in an aqueous solution of imidacloprid on exposure to natural sunlight [40,41]. After irradiating the solution of imidacloprid under natural sunlight for 150 min, the absorbance of the solution was measured at (λ_max_ 269.5 nm) using a UV–Visible spectrophotometer (CE Cecil 7200, Isernhagen, Germany). Finally, the % degradation of imidacloprid using blank and polyester grafted as-fabricated and prewetted samples of nanocalcite @ PF was calculated and compared using the following equation [42].

(1) $$\%\;\mathrm{degradation}=\frac{\mathrm{initial}\;\mathrm{absorbance}-\;\mathrm{final}\;\mathrm{absorbance}}{\mathrm{Initial}\;\mathrm{absorbance}}\times 100$$

The extent of photocatalytic degradation of the imidacloprid solution was also analyzed by comparing the results of the as-fabricated and treated solutions.

## 3. Results and Discussion

### 3.1. Characterization of Nanocalcite @ PF

The analysis of the as-fabricated and prewetted nanocalcite @ PF was performed and compared for crystallinity, morphology, surface charge, and optical properties.

#### 3.1.1. XRD Analysis of Nanocalcite @ PF

**Figure 2:**
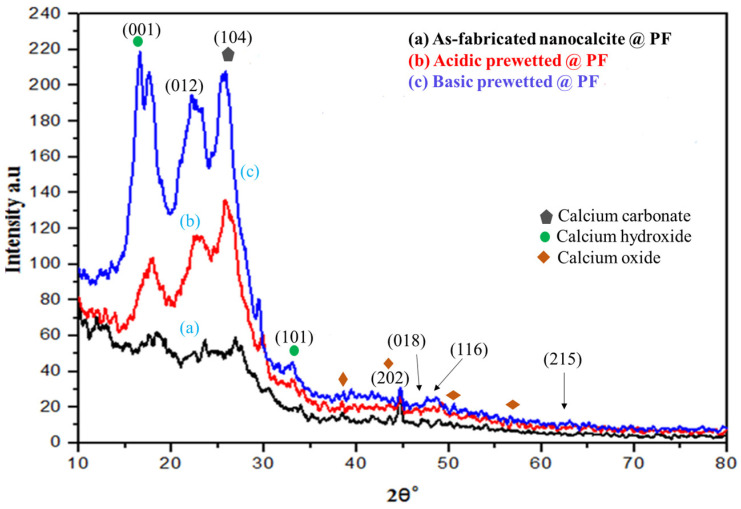
XRD of (**a**) as-fabricated nanocalcite @ PF, (**b**) acidic prewetted nanocalcite @ PF, (**c**) basic prewetted nanocalcite @ PF.

Diffractograms of the surface grafted nanocalcite @ PF of the as-fabricated and prewetted samples are shown in Figure 2. The XRD peaks appeared at 2θ° value of 18.67° (001), 23.1° (012), 29.3° (104), 33.96° (101), 43.1° (202), 47.1° (018), and 47.5° (116). The diffraction peaks appeared at 2θ° = 23.1° (012), 29.3° (104), 43.1° (202), 47.1° (018), 47.5° (116) were in very close agreement with the standard JCPDS file (JCPDS-05-0586) for nanocalcite. The peak appearing at 29.3° (104) is representative of the crystal face belonging to calcite [43]. The peaks in addition to nanocalcite that appeared at the 2θ° value of 18.67° and 33.96° were for calcium hydroxide and 37.39°, 47.5°, and 51.3° for calcium oxide [44]. After prewetting, the peak intensity increased, which exhibited improved crystallinity. Furthermore, analysis of the diffractogram by using Xpert-Highscore and Match showed that nanolime (Ca(OH)_2_ and nanocalcite (CaCO_3_) along with some CaO were present in the as-fabricated nanocalcite @ PF, but nanocalcite was considered as the main component of the mixture by Match software. Nanocalcite has a rhombohedral crystal structure [45,46,47]. Calcite with space group R3c having rhombohedral crystal was found to show angles α = β = 90° and γ = 120° between edges a = b = 4.880 Å and c = 17.061 Å [43]. The average crystallite size of the anocalcite was derived by the Debye–Scherer’s Equation [48] (Equation (2)).

(2) $$L=\frac{k\lambda }{\beta \cos\theta }$$

where the wavelength of X-rays using (CuKα; λ = 1.54 Å), k = 0.9, θ=2θ/2 (from diffractogram), β = FWHM (from diffractogram); *L* represents average crystallite size (nm); β is the line broadening; and θ is a Bragg’s angle. Using the peak intensity at (104), for an average grain size of the as-fabricated, acidic and basic prewetted nanocalcite @ PF was 46.76 nm, 39.43 nm, and 32.26 nm, respectively.

#### 3.1.2. FTIR Analysis of Nanocalcite @ PF

**Figure 3:**
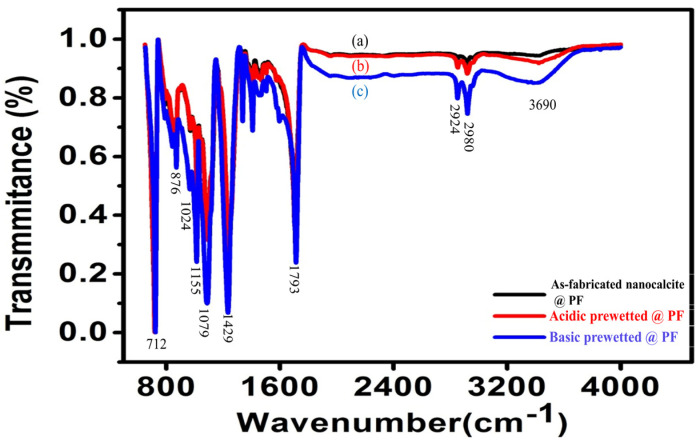
Normalized FTIR spectrum of (**a**) as-fabricated nanocalcite @ PF, (**b**) acidic prewetted nanocalcite @ PF, and (**c**) basic prewetted nanocalcite @ PF.

A change in material composition is indicated by a change in a characteristic pattern of absorption bands of stretching and banding vibrations in the IR region and is shown in Figure 3a–c. In the sample, a narrow absorption band at 3690 cm^−1^ was due to the stretching mode of the oxygen–hydrogen present. The absorption bands at 1464, 1079, and 876 cm^−1^ were given due to different vibration modes of the carbon-oxygen of the carbonate groups. Additionally, there was a tiny dip in the spectra at 2924 cm^−1^ due to the adsorbed gaseous carbon dioxide, as indicated in previous research [49,50,51]. All calcium carbonate samples showed peaks at ~1793, 1429, 1024, 876, and 712 cm^−1^, respectively, assigned to the asymmetric vibration of the carbon–oxygen bond, which are also in alignment with the previous reports [46,52]. The increment in the intensity of the most characteristic groups due to the oxidation effect caused by photochemical interactions and polymer degradation is clear. In the spectrum of the basic prewetted treatment of nanocalcite @ PF in Figure 3c, the sharp absorption band with a high peak intensity at 3690 cm^−1^ is a characteristic of the hydroxyl group, which revealed the presence of additional hydroxyl groups. This increase in adsorption intensity indicated the introduction of acidic groups. Besides this, a slight decrease in adsorption intensity at 1429 cm^−1^ was observed in Figure 3c relative to Figure 3a,b due to the bending vibrations of the carbon–hydrogen bond in the methylene group. Conclusively, the proportion of oxygen-containing groups such as carboxyl, carbonyl, and alcohol groups increased on the prewetted surface of nanocalcite @ PF. An increase in the oxygen-containing groups on the surface of @ PF modified the surface of the fabric from hydrophobic to hydrophilic, which has already been reported [6]. Hence, it was observed from the comparative peak area of the hydroxyl group that the density of the hydroxyl group onto the nanocalcite @ PF surface highly increased in the basic prewetting treatment (pH = 11).

#### 3.1.3. Scanning Electron Microscopic Analysis (SEM) of Nanocalcite @ PF

**Figure 4:**
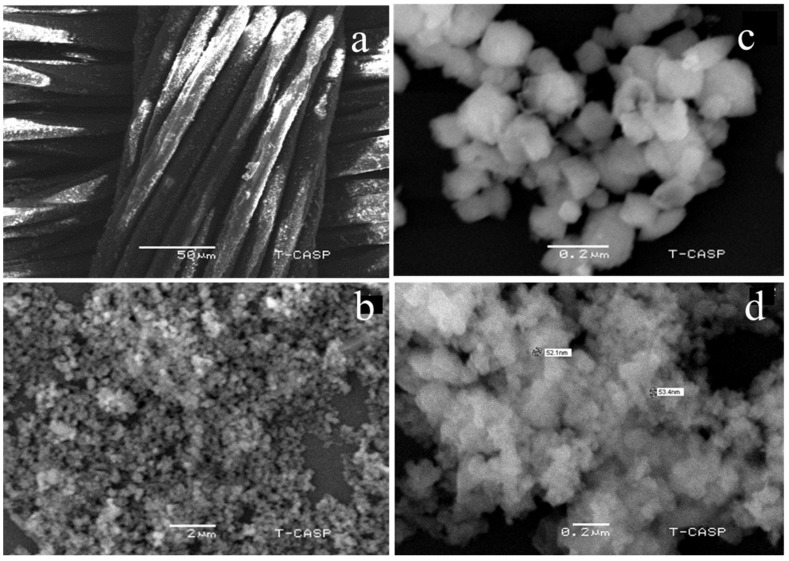
Micrographs of (**a**) Functionalized polyester with grafted nanocalcite at 10KX, (**b**) As-fabricated nanocalcite @ PF, (**c**) acidic prewetted nanocalcite @ PF, (**d**) basic prewetted nanocalcite @ PF.

The change in the morphological structure of nanocalcite @ PF due to prewetting treatment was investigated by comparing the as-fabricated and prewetted materials as shown in Figure 4a–d. It is clear from the micrograph of the surface-functionalized polyester loaded with the as-fabricated nanocalcite that the surface was fully covered with dense monomodal nanocalcite particles (Figure 4a). The discoid structure of nanocalcite showed a smooth contour adhered onto the surface of PF (Figure 4b). On acid prewetting of nanocalcite @ PF, the surface roughness due to kinks and corners appeared (Figure 4c). Similar results were obtained by other researchers on acid prewetting [53]. Upon basic prewetting surface roughness increased causing the appearance of fluffy and eroded boundaries of discs (Figure 4d). The basic prewetted nanocalcite grafted onto the surface of the polyester fabric exhibited enhanced nanoscale surface roughness due to the excessive induction of hydroxyl groups. Moreover, on the basic pretreated surface, the fine kinks and grooves appeared due to significant erosion on the surface, which were distinguishable in micrograph (Figure 4c,d). This surface roughening effect leads to enhanced light-trapping phenomenon due to destructive interference [54,55]. The surface roughness of nanocalcite @ PF increased the surface area of the fabric due to basic prewetting due to the greater extent of surface erosion occurred compared to that caused by acidic prewetting. Moreover, it was observed that surface roughness increased with the concentration in the basic solution used for pretreatment, indicating that the surface undergoes hydrolysis, which enhanced the surface hydrophilicity. Such effects of prewetting have been reported previously, which support the present results [53,55]. It can be seen that the as-fabricated surface looked very smooth compared to that of the pretreated one, which shows that the roughness was because of partial hydrolysis. Distinguishably small pits were present on the pretreated surface (Figure 4).

#### 3.1.4. Optical Properties of As-Fabricated and Basic Prewetted Nanocalcite @ PF

**Figure 5:**
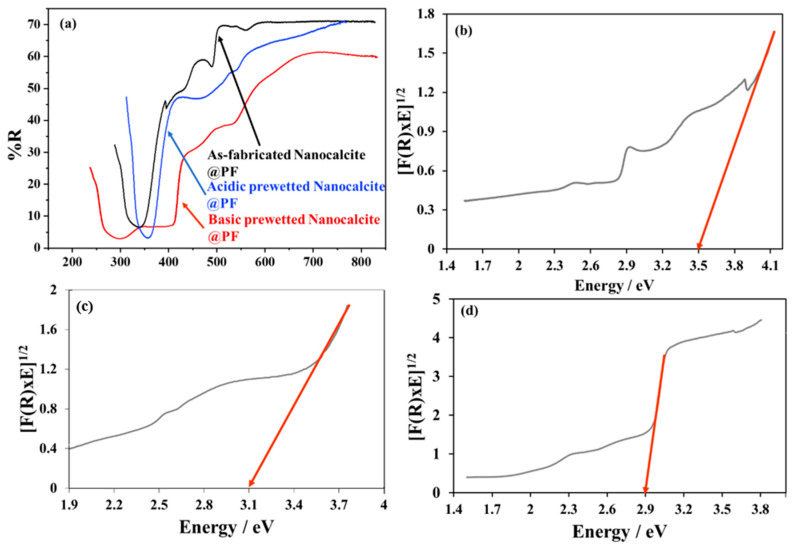
(**a**) Comparative diffused reflectance spectra of the as-fabricated and basic prewetted nanocalcite @ PF, (**b**) band gap of as-fabricated nanocalcite @ PF, (**c**) band gap of acidic prewetted nanocalcite @ PF, and (**d**) band gap of basic prewetted nanocalcite @ PF.

The comparative spectral study of the as-fabricated and basic prewetting delineated that with basic prewetting, the material harvested a higher percentage (60–70%) of solar radiation below 500 nm and the bandgap edge was shifted to 410 nm in comparison to the bandgap edge of the as-fabricated nanocalcite @ PF at 350 nm (Figure 5a). The enhanced harvesting capability of basic prewetted nanocalcite @ PF can be attributed to the surface erosion of functionalized fabric and induction of hydroxyl groups on the surface. The bandgap of nanocalcite @ PF was calculated by the Kubelka Munk plot by using the data obtained from diffused reflectance spectroscopy. The bandgap of the as-fabricated nanocalcite @ PF determined was 3.5 eV (Figure 5b), basic prewetted nanocalcite @ PF was reduced to 2.9 eV (Figure 5c) while that with acid prewetted was shifted to 3.1 eV at 410 nm on the absorbance edge, falling in the visible range of the solar spectrum. Decrease in bandgap due to surface roughness created defects on the surface and interband energy states ultimately appeared below the conduction band. This attribution for the creation of interband structures has also been expressed in other research reports [17,56,57]. Subsequently, electrons require less energy to be excited from the valence band (VB) to the conduction band (CB).

### 3.2. Evaluation of Hydrophilicity in Nanocalcite @ PF on Prewetting

The extent of hydrophilicity of the as-fabricated and prewetted surfaces were investigated by contact angle measurement and wickability testing.

#### 3.2.1. Contact Angle Measurement of Nanocalcite @ PF

**Figure 6:**
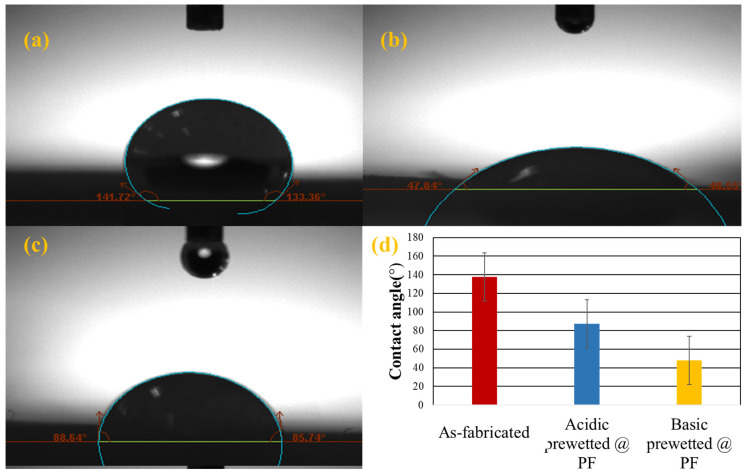
The average water contact angle of the (**a**) as-fabricated nanocalcite @ PF, (**b**) basic prewetted nanocalcite @ PF, (**c**) acidic prewetted nanocalcite @ PF, and (**d**) comparison of the contact angle value of the as-fabricated and prewetted nanocalcite @ PF.

To determine the wettability, the contact angle between the surface of the solid and water droplet was determined using optical tensiometers Water droplets placed on the surface grafted nanocalcite @ PF by using micro-syringe and the contact angle were measured by optical tensiometer using the sessile drop method. If the water droplet spreads more on the surface and the contact angle is less than 90°, the surface is considered to be hydrophilic [58,59]. The contact angles of the as-fabricated, acidic and basic prewetted nanocalcite @ PF swatch samples are given in Table 2 and Figure 6a–c.

#### 3.2.2. Wickability Analysis of Nanocalcite @ PF

The wicking test is a spontaneous displacement of the solid/liquid interface in the capillary system. The extent of wicking within the fabric depends on the surface hydrophilic group, pore distribution, and pore size [33]. Conventionally, to enhance the hydrophilicity of the fabric, alkaline pretreatments were applied using potassium hydroxide. During such treatments, the hydroxyl group is formed on the breakage of the hydroxyl bond by prewetting treatment, which improves fiber hydrophilicity [60].

**Figure 7:**
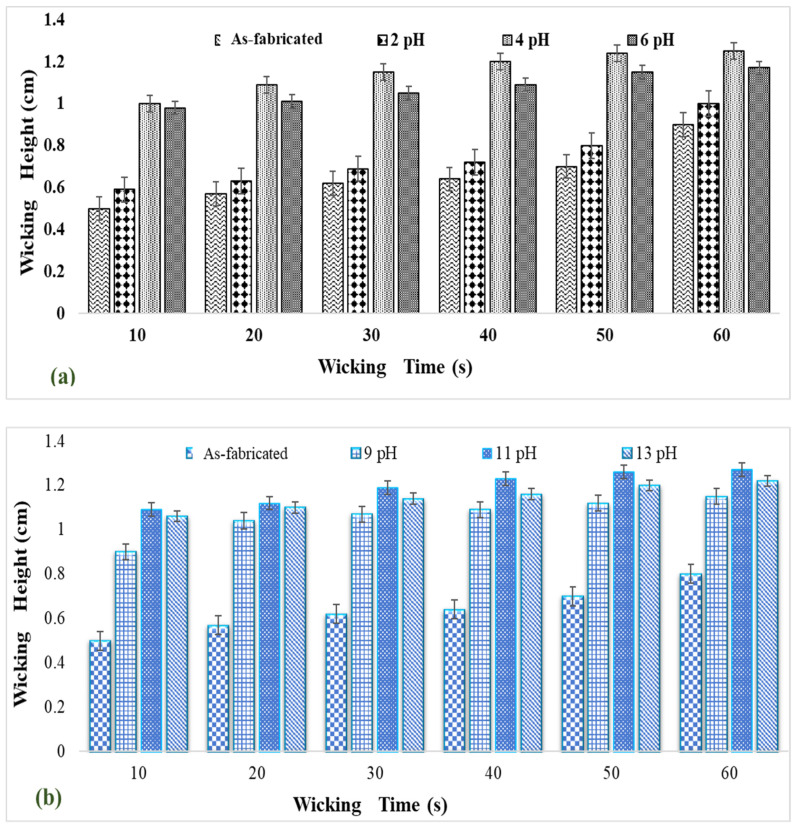
Wickability of (**a**) acidic prewetted nanocalcite @ PF and (**b**) basic prewetted nanocalcite @ PF in water.

Wickability data on the surface grafted nanocalcite @ PF in water prewetted with the acid and base are given in Figure 7a,b. The swatches of surface grafted nanocalcite @ PF (5 × 5 cm^2^) treated for acid prewetting (pH = 4) and basic prewetting (pH = 11) exhibited a high wicking rate.

#### 3.2.3. Determination of Surface Charge of Nanocalcite @ PF by Zeta Potential

With the enhancement of the Zeta potential value on acid and basic prewetting, the surface charge produced by basic prewetted (0.721 mS/cm) was greater compared to the acidic prewetted (0.552 mS/cm) nanocalcite @ PF. The relative charge density of surface depends on the relative concentration of the hydroxyl ion and hydrogen ion, which behave as potential determining ions. Early work suggested that the Zeta potential of the calcite material is due to the presence of hydrogen ions, calcium ions, hydroxyl ions, carbonate, and bicarbonate ions on the surface [61]. The high positive or negative value of the Zeta potential indicates less agglomeration (i.e., more surface grafted nanocalcite @ PF particles were available onto the surface of the polyester substrate) [62,63]. The Zeta potential depends on the experimental condition, calcite nature, method of preparation, and measurements [64,65].

**Figure 8:**
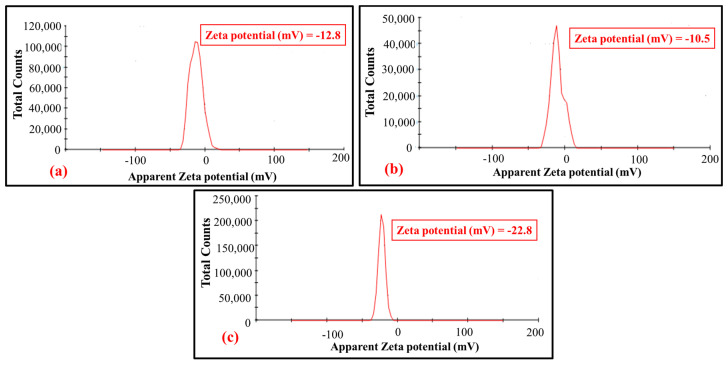
Zeta potential of the (**a**) as-fabricated nanocalcite @ PF, (**b**) acidic prewetted nanocalcite @ PF, (**c**) basic prewetted nanocalcite @ PF.

The difference in the surface charge of nanocalcite @ PF in an aqueous dispersion could be explained based on surface basicity and acidity or the difference in ionic species that were adsorbed on the surface of nanocalcite @ PF [66,67]. The variation in the Zeta potential of nanocalcite @ PF (−12.8 to −22.8 mV) was shown in Figure 8a–c. The observed value of 12.8 mV for the as-fabricated nanocalcite @ PF, 10.5 mV for acid prewetted (pH = 4), and 22.8 mV for basic prewetted (pH = 11) in their aqueous suspension. When nanocalcite was dispersed in water, then the surface of PF became anionic and an increase in surface charge occurred, which caused denser coverage of hydroxyl groups from the water and generated fewer hydrogen ions [68]. Zeta potential of the as-fabricated, acidic, and basic prewetted nanocalcite @ PF became negative. Moreover, Zeta potential of surface grafted nanocalcite @ PF was found to be associated with the surface conductivity of the materials. Zeta potential and conductivity of the as-fabricated and prewetted surface grafted nanocalcite @ PF after acidic and basic prewetting pH treatments is shown in Figure 8a–c.

### 3.3. Photocatalytic Degradation of Imidacloprid (Insecticide) Using Nanocalcite @ PF

The characterized surface grafted nanocalcite @ PF was utilized for the photodegradation of imidacloprid on irradiating the reaction mixture to natural sunlight. Response surface methodology has many advantages such as model fitting for the optimization of variables, cost-effectiveness, and time efficiency [26,69]. In the present study, 20 runs with various combinations of X_1_, which represented irradiation hours under natural sunlight, X_2_ as the pH level, and X_3_ as the oxidant (hydrogen peroxide) concentration in mM; the response of the three parameters are given in Table 3. The results show the maximum efficiency of % degradation of imidacloprid solution treated with basic prewetted pH nanocalcite @ PF. The maximum % degradation efficiency of the insecticide solution was achieved in run numbers 2, 6, 8, 12, 13, and 18 attained at pH 11 (reaction solution pH), oxidant concentration was 30 mM, and irradiation time was three hours. On the other hand, minimum degradation was observed in run number 3 at pH 13, oxidant concentration was 50 mM, and irradiation time 5 h.

#### 3.3.1. Photocatalytic Activity of Basic Prewetted Nanocalcite @ PF

**Figure 9:**
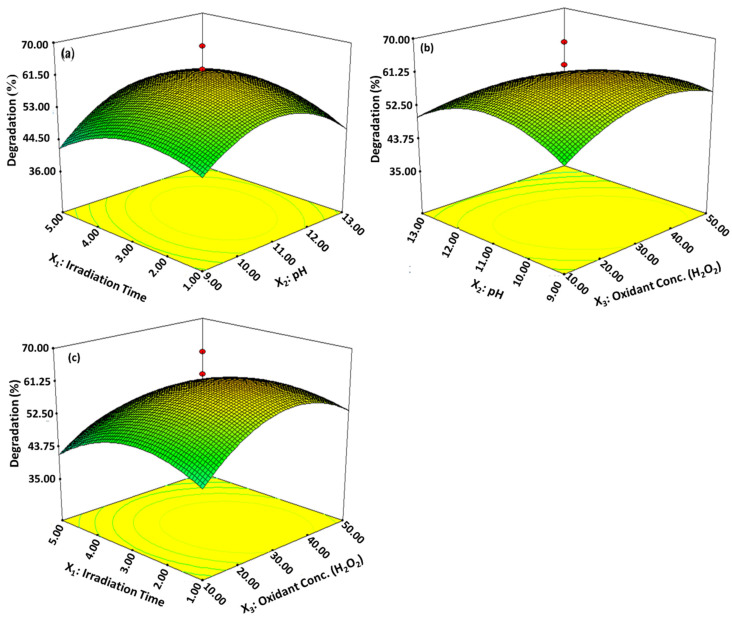
3D response surface plots. (**a**) Irradiation time and pH, (**b**) pH and concentration of oxidant, (**c**) Irradiation time and concentration of the oxidant, respectively, showing the interactive effect of the independent variable of % degradation of imidacloprid treated with basic prewetted pH on nanocalcite @ PF.

The effects of variable interaction were identified as well as their ideal values by plotting the curve of a response surface for combination between the variables X_1_ and X_2_, X_3_ and X_2_ and X_1_ and X_3_, while % degradation of imidacloprid solution was plotted on the *Z*-axis [70]. It is shown in Figure 8 by the response surface plots and their interactive effects among the operational parameters occurred. Figure 9a represents the interaction between the irradiation time (3 h) and initial pH level 11 (reaction solution pH), while the third parameter remained constant and the maximum degradation of the imidacloprid solution was 60.14%. It was observed that the maximum degradation was obtained at alkaline pH (reaction solution pH). Maximum degradation of 63.34% (Figure 9b) shows that the 3D surface plot between pH 11 (reaction solution pH) and the concentration of hydrogen peroxide was 30 mM. Figure 9c shows that the maximum photodegradation of insecticide was 69.21%, obtained at an irradiation time of 3 h and 30 mM of oxidant concentration while it decreased with increasing pH from 9 to 11. It has been shown that the rate of degradation of the insecticide accelerated at pH 11 (alkaline pH), and a further increase in pH degradation rate became slower. The irradiation time is an important factor that plays a vital role in the degradation rate of the insecticide. As the irradiation time increased, the extent of photodegradation was magnified because more photons were available, which produced a higher number of electron hole pairs. The extent of degradation increased from 1 to 3 h and after that, no significant change occurred due the limitation caused by the solar energy harvested and recombination of electron and hole pairs. Rate of photocatalytic degradation of imidacloprid was increased with elevated hydrogen peroxide concentration and aided in situ generation of ^●^OH radicals. The degradation rate was increased with a hydrogen peroxide concentration up to 30 mM, however, a further increase in the concentration of the oxidant beyond the optimum level retarded the degradation rate, even when increasing the irradiation time [17]. Under an optimized reaction condition, the % degradation of imidacloprid was 91.49% for basic prewetted nanocalcite @ PF in 150 min.

#### 3.3.2. Statistical Analysis for As-Fabricated and Basic Prewetted Nanocalcite @ PF

Statistical analysis data are represented in Table 4, where R^2^ (determination coefficient) of the model was 0.9148, evidence that 69.21% degradations were attributed to the independent variable. An R^2^ value of the regression model higher than 0.9 was considered a very high correlation [70]. The perfect compatibility between the predicted and the observed value of degradation by the response agreement between the “Adj R-squared” of 0.9148, lower C.V. value of 9.28%, and standard deviation of 4.59, respectively [71,72]. The model F-value of 11.93604 with a low probability value (*p*-value 0.0003) implies that the model was significant. In Table 4, the significance degree indicated that linear coefficients of X_1_, X_2_, and X_3_ and the quadratic effect of three independent variables were the significant models [73]. Using a second-order polynomial equation that determines the response (Y) obtained in the term of independent factors (Equation (3)):

Y = + 61.42 + 1.76A − 0.85B − 4.25C + 1.91AB − 1.97AC − 4.09BC − 8.90A^2^ − 5.64B^2^ − 6.74C (3)

where Y is the % degradation efficiency of the insecticide solution (the response) and X_1_, X_2_ and X_3_ are the irradiation hours, pH value, and oxidant concentration, respectively.

### 3.4. Evaluation of Extent of Photocatalytic Degradation of Imidacloprid Solution

**Figure 10:**
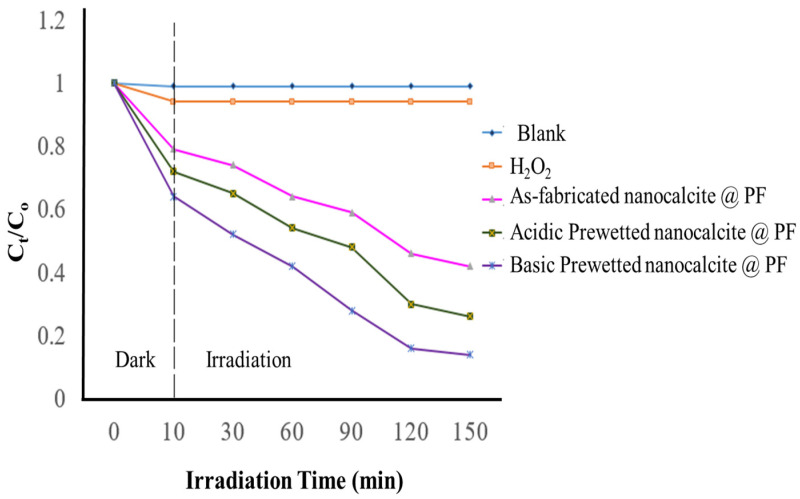
Determination of the extent of photocatalytic degradation (C_t_/C_o_) of imidacloprid (C_o_ = 100 mg/L, concentration of hydrogen peroxide 10 mM, initial pH 7.0, (irradiation time) under natural sunlight for 150 min by using nanocalcite @ PF with respect to time.

The degradation extent of the imidacloprid solution was compared with the as-fabricated and basic prewetted (pH 11) nanocalcite @ PF submerged in 30 ppm solution of insecticide (imidacloprid). Under optimized conditions of pH 11 using 30 mM concentration of oxidant (hydrogen peroxide), maximum degradation was obtained upon irradiating for 3 h under natural sunlight as given in Figure 9. In the graph of C_t_/C_o_ for the as-fabricated, oxidant bearing and treated solution of imidacloprid (λ_max_ = 269.5 nm) by the as-fabricated and basic prewetted nanocalcite @ PF were compared. It is obvious from the graph that there was no decrease in C_t_/C_o_ concerning time for the as-fabricated solution and almost the same trend was shown on the addition of only hydrogen peroxide (as the control). The minimum C_t_/C_o_ value for imidacloprid (30 ppm, 100 mL) was obtained using basic prewetted nanocalcite @ PF. The initial magnificent decrease in C_t_/C_o_ was attributed to adsorption of the pollutant by the photocatalyst in the dark, which further decreased on the irradiating solution under natural sunlight. However, the photocatalytic degradation of imidacloprid using as-fabricated nanocalcite @ PF was less compared to the acidic prewetted (pH 4) and basic prewetted (pH 11) nanocalcite @ PF, which showed maximum degradation of 82.28% and 91.49%, respectively, in 150 min, as shown in Figure 10.

The acidic and basic prewetted treatments enhanced the hydrophilicity of the surface grafted nanocalcite @ PF. The surface hydrophilicity changed the molecular chemical reaction at the surface of the nanocalcite @ PF due to increased polarity and modified surface chemistry [74].

In the process of photocatalytic degradation, photocatalyst used was semiconductor metallic oxide. When the surface of the semiconductor metallic oxide was irradiated by solar light, it started the redox reaction. The bandgap of semiconductors is associated with the space between energy levels (i.e., valence and conduction bands). Photoexcitation with an energy greater or equal to that energy gap between the conduction and valance bands moves an e^−^ from the valance band to the conduction band, generating a hydrogen ion deficiency in the electron. The oxidation of an adsorbed molecule can occur by reacting with h^+^, and simultaneously, the reduction of another molecule can take place with e^−^ present in the conduction band of the photocatalyst [75]. In photocatalytic treatments, the concentration of H_2_O_2_ produced after the solar photocatalytic reaction affects the extent of photodegradation via a modification due to adsorption of hydrogen peroxide onto the surface of the photocatalyst [76,77]. The inhibition of holes produced in the valance band was observed due to their reaction with hydroxyl radicals. However, a small amount of hydrogen peroxide was found to be useful as the optimum consumption of hydrogen peroxide is essential in order to obtain only a small augmentation in degradation extent [78].

The decrease in the contact angle values and surface roughness increased with the large number of surface adhered hydroxyl groups that showed remarkable hydrophilic properties. It can be concluded that surface roughness is the main factor, which renders the surface hydrophilic. The hydrophilic property of the material surface is characterized by the water contact angle measurements. The most common defect on the surface of heterogeneous catalysis is oxygen vacancies (OVs), which showed the ability of the dissociating hydrogen peroxide on the surface of the nanocatalyst to generate hydroxyl (^●^OH) radicals. The result indicated that many metal oxide catalysts with abundant OVs on the surface could accelerate hydroxyl (^●^OH) radical generation and further improve the catalytic activity of the heterogeneous photocatalysts [79]. When water droplets are placed on the surface, the water molecules occupy oxygen vacancies and the hydrophilic hydroxyl group is absorbed on the surface, which tends to turn the surface more hydrophilic. Generally, a variety of oxygen-containing groups such as carboxyls, carbonyls, and hydroxyls are introduced onto the surface of the material, which increases the surface energy [80]. Moreover, roughness and dangling bonds enhance the hydrophilic property of the surface. When the crystal structure is disrupted (i.e., at a grain boundary and a surface), dangling bonds are formed [81]. The nanometer-scale surface roughness and dangling bond on the surface of the fabric improved the surface energy of the liquid–solid interface and thus wetting of the surface of the nanocalcite @ PF [82].

#### 3.4.1. Evaluation Degradation of Imidacloprid by UV–Visible Spectroscopy

**Figure 11:**
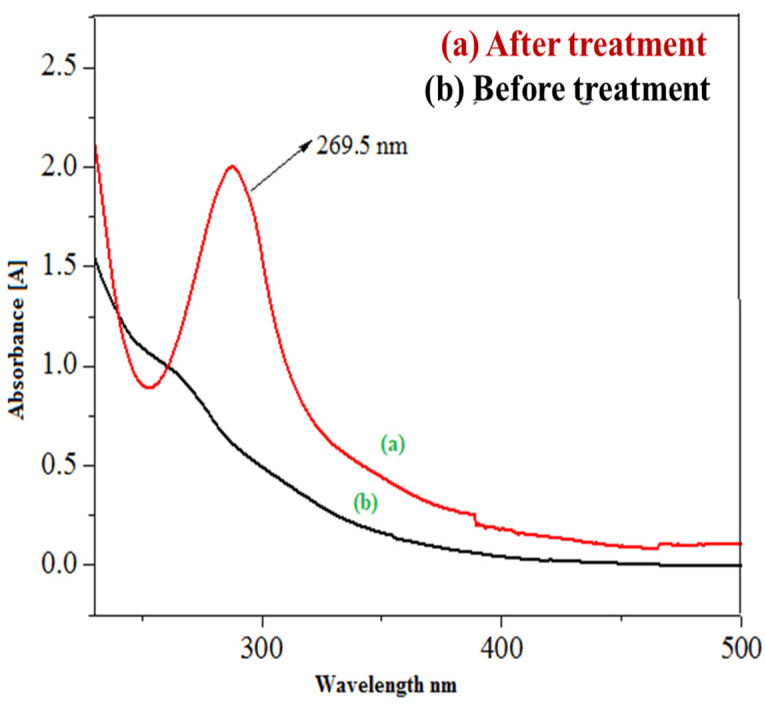
UV/Vis absorbance spectra of (**a**) as-fabricated imidacloprid sample and (**b**) sample of imidacloprid treated with basic prewetted nanocalcite (pH 11).

Since the basic prewetted sample exhibited the leading photocatalytic degradation of imidacloprid, the sample treated by the basic prewetted nanocalcite @ PF was analyzed by UV–Visible spectroscopy (Figure 11). The water sample containing insecticide (imidacloprid: 30 ppm) was analyzed by UV–Visible spectrophotometry (CE Cecil 7200, Germany) at the Radiation Chemistry Laboratory, University of Agriculture, Faisalabad. The decomposed samples of imidacloprid using basic prewetted nanocalcite @ PF were also measured. The absorbance of as-fabricated imidacloprid solution was 1.882 and the solution treated with basic prewetted nanocalcite @ PF (pH = 11) was 0.16 after exposure to natural sunlight for 150 min [83].

#### 3.4.2. High Performance Liquid Chromatographic Analysis of Imidacloprid

**Figure 12:**
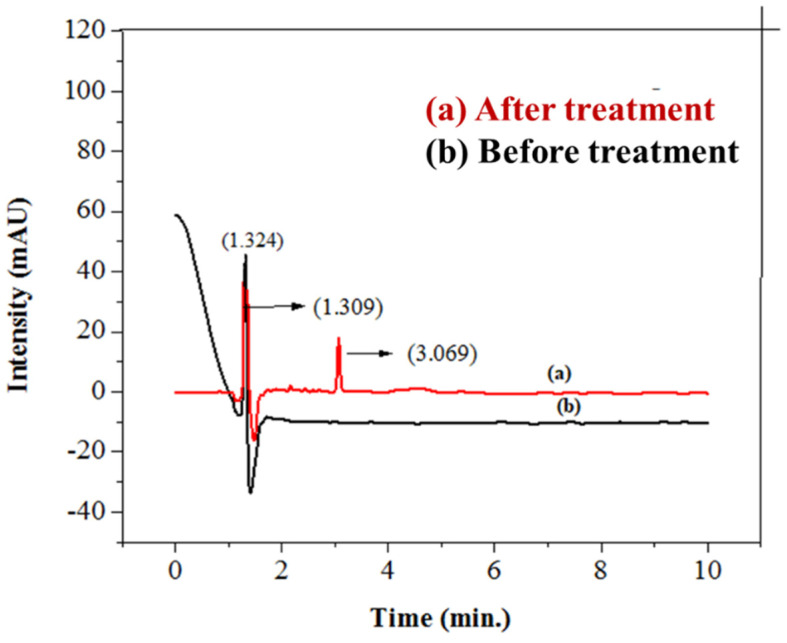
HPLC chromatogram of (**a**) sample of imidacloprid treated with basic prewetted nanocalcite @ PF (pH 11) and (**b**) untreated imidacloprid sample.

To further investigate the photocatalytic degradation of imidacloprid, HPLC was employed using a 20 µL injection of imidacloprid. A shift in peak position, decrease in peak height, and peak area of insecticide in wastewater was observed, as indicated in previous reports [84]. Due to the π–π* of the nitroguanidine chromophore, the studied wavelength of imidacloprid (270 nm) was found to be the best for the detection of the imidacloprid solution in terms of height or peak area [85]. After treatment, the height, area, and retention time of imidacloprid decreased due to its degradation in the smallest innocuous hydrocarbons [86]. Before treatment, the peak of imidacloprid (30 ppm, 20 µL) appeared at a height of 86.23747 mAU, having an area of 552.72260 mAU*s and retention time of 1.721 min. After treating the imidacloprid solution with basic prewetted nanocalcite @ PF, the peak intensity was decreased, having a peak, area, and retention time of 327.79218 mAU, 327.79218 mAU*s, and 1.309 min, as given in Table 5. The results clearly show a shift in the peak position of pesticide after treatment with a photocatalyst, as shown in Figure 12.

#### 3.4.3. FTIR Spectrum Analysis of As-Fabricated and Prewetted Imidacloprid Solutions

**Figure 13:**
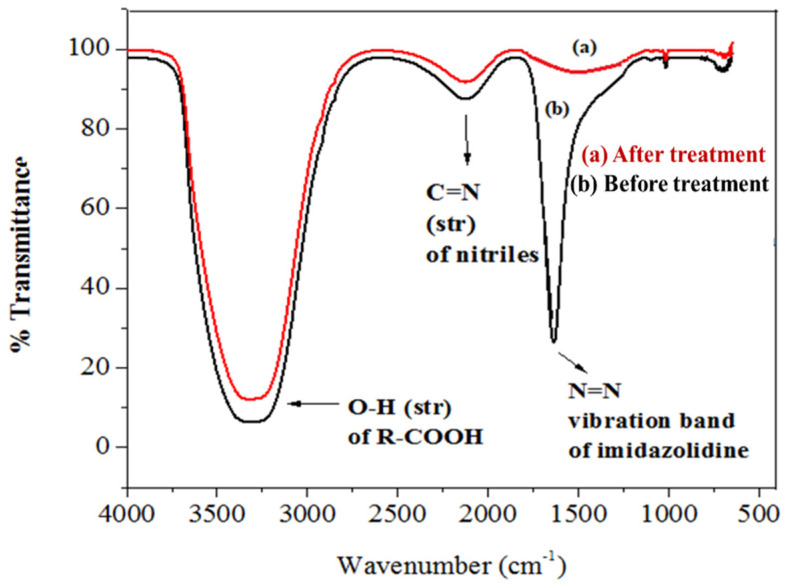
FTIR spectrum of the (**a**) sample of imidacloprid treated with basic prewetted (pH 11) and (**b**) as-fabricated untreated imidacloprid sample.

**Figure 14:**
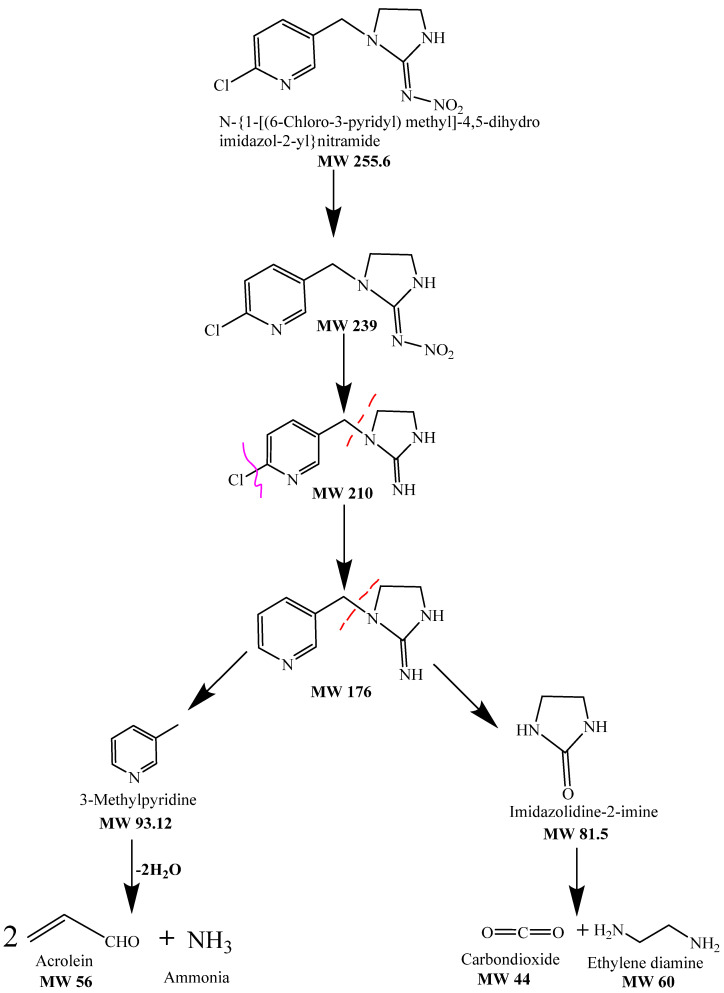
Degradation mechanism of imidacloprid degradation using basic prewetted nanocalcite @ PF.

FTIR spectrum of the control displayed peaks at 1566.25 cm^−1^ for the vibration band of N=N in the imidazolidine ring of imidacloprid and at 1240.27 cm^−1^ for the vibration band of C=N in the pyridine ring. The spectrum of FTIR of the samples’ peaks at 3128.49 cm^−1^ for the O–H stretching of the R-carboxylic acid group [87,88]. Figure 13 shows the FTIR spectrum for the treated and untreated imidacloprid solution (30 ppm). The solar photocatalytic degradation mechanism of imidacloprid is shown in Figure 14. In the presence of basic prewetted nanocalcite @ PF upon solar irradiation at optimized condition, the imidacloprid ring decomposed into different fragments and converted into innocuous compounds as end products.

## 4. Conclusions

In the present research, nanocalcite was grafted onto the surface of pretreated polyester fabric. The process of seeding was followed by the growth of nanocalcite for the functionalization of the fabric. The fabricated solar photocatalytic reactor was prewetted with acids and bases for surface modification. The as-fabricated functionalized PF and its prewetted samples were characterized for the crystallinity of nanocalcite, its composition, morphology, and optical properties. The basic prewetted nanocalcite @ PF was found to have higher sunlight harvesting capability due the decrease in bandgap (2.9 eV) compared to the as-fabricated and acidic prewetted solar photocatalytic reactors. The surface charge was also measured to be 12.8 mV for the as-fabricated nanocalcite @ PF, 10.5 mV for acid prewetted (pH = 4), and 22.8 mV for basic prewetted (pH = 11). The contact angles of the as-fabricated nanocalcite @ PF sample was determined to be 137.54°, while it reduced to 87.17° and 48.19° for acidic and basic prewetted samples, exhibiting the highest hydrophilicity of basic prewetted nanocalcite @ PF. The wicking test of all samples also supported the results of the contact angle measurement. The photocatalytic degradation of the toxic insecticide imidacloprid was studied using nanocalcite @ PF, acidic prewetted nanocalcite @ PF, and basic prewetted nanocalcite @ PF. Reaction process parameters; pH 11, oxidant concentration 30 mM, and sunlight irradiation of 3 h was optimized using response surface methodology. The maximum % degradation of imidacloprid using control, acidic prewetted, and basic prewetted nanocalcite @ PF was 70.68%, 82.28%, and 91.49%, respectively, in 3 h. The surface prewetting of nanocalcite grafted onto polyester fabric at pH 11 can render the photocatalyst more hydrophilic with enhanced photocatalytic activity. The proposed method of prewetting has been experienced to be convenient and efficient for the enhancement of the photocatalytic activity of an immobilized semiconductor nanocalcite.

## Figures and Tables

**Table 1:** Coded reaction parameters and their level ranges investigated for optimization by RSM.

Factor	Variables	Units	Low Actual	High Actual
X_1_	Sunlight	Hours (h)	1	5
X_2_	pH	---	9	13
X_3_	Oxidant Concentration of H_2_O_2_	mM	10	50

**Table 2:** Alteration of the water contact angle of the as-fabricated and after acidic and basic prewetted nanocalcite @ PF.

Functionalized Polyester Fabric	Angles between Solid Surface of Nanocalcite @ PF and Liquid Surface		Average of Angles between Solid Surface of Nanocalcite @ PF and Liquid Surface
As-fabricated	θ_1_	θ_2_	θ1+θ22
	141.72°	133.36°	137.54°
Basic prewetted	47.84°	48.55°	48.195°
Acidic prewetted	88.64°	85.74°	87.19°

**Table 3:** Central composite design with variable values and percent degradation of imidacloprid.

Run	X_1_	X_2_	X_3_	Degradation (%)
1.	1.00	13.00	10	43.1
2.	3.00	11.00	30.00	60.14
3.	5.00	13.00	50.00	32
4.	3.00	14.00	30.00	42.4
5.	0.36	11.00	30.00	42.07
6.	3.00	11.00	30.00	60.14
7.	3.00	11.00	63.64	35.66
8.	3.00	11.00	30.00	60.22
9.	1.00	9.00	50.00	39.65
10.	1.00	9.00	10.00	42.39
11.	5.00	13.00	10.00	58.98
12.	3.00	11.00	30.00	60.14
13.	3.00	11.00	30.00	63.34
14.	5.00	9.00	50.00	40
15.	1.00	13.00	50.00	32.2
16.	5.00	9.00	10.00	42.44
17.	3.00	11.00	3.64	49
18.	3.00	11.00	30.00	69.21
19.	6.36	11.00	30.00	36.76
20.	3.00	7.64	30.00	47.5

**Table 4:** ANOVA for face-centered composite design experimental value of % age degradation efficiency of the insecticide solution treated with basic prewetted pH on nanocalcite @ PF.

Source	Sum of Squares	d.f	Mean Square	F Value	*p*-Value Prob > F	
Model	2258.737	9	250.9708	11.93604	0.0003	significant
Irradiation Time (X_1_)	37.28524	1	37.28524	1.773267	0.2125	
pH (X_2_)	9.351642	1	9.351642	0.444759	0.5199	
Oxidant Conc.(X_3_)	218.3313	1	218.3313	10.38372	0.0091	
(X_1_, X_2_)	29.1848	1	29.1848	1.388014	0.2660	
(X_1_, X_3_)	31.12605	1	31.12605	1.480339	0.2517	
(X_2_, X_3_)	133.6613	1	133.6613	6.356861	0.0303	
X_1_^2^	848.4032	1	848.4032	40.34962	<0.0001	
X_2_^2^	393.8509	1	393.8509	18.73135	0.0015	
X_3_^2^	487.3713	1	487.3713	23.17913	0.0007	
Residual	210.263	10	21.0263			
Lack of Fit	143.1721	5	28.63442	2.134002	0.2126	not significant
Pure Error	67.09088	5	13.41818			
Core total	2469	19				
Std Dev. = 4.59	C.V. = 9.28%		R^2^ = 0.9148		Adj R^2^ = 0.8382	

C.V: Coefficient of variation, d.f: Degree of freedom, P: Level of significance, F: Fishers’s function.

**Table 5:** Data analysis of the HPLC chromatogram for the imidacloprid solution treated with basic prewetted nanocalcite @ PF.

Peak #	Ret.Time (min.)	Type	Width (min.)	Area (mAU*s)	Height (mAU)	Area %
Before Deg.	1.721	BB	0.0583	552.72260	86.23747	62.7727
After Deg.	1.309	BB	0.3846	327.79218	17.59007	37.2273

## Data Availability

The data that support the findings of this study are available from the corresponding author upon reasonable request.
